# The effects of vitamin D and calcium on primary dysmenorrhea: a systematic review

**DOI:** 10.25122/jml-2023-0248

**Published:** 2023-11

**Authors:** Ainur Donayeva, Ainur Amanzholkyzy, Ibrahim Abdelazim, Samat Saparbayev, Roza Nurgaliyeva, Aiman Kaldybayeva, Azhar Zhexenova, Gulnara Gubasheva, Dinmukhamed Ayaganov, Ihab Samaha

**Affiliations:** 1 Department of Normal Physiology, West Kazakhstan Marat Ospanov Medical University, Aktobe, Kazakhstan; 2 Department of Obstetrics and Gynecology, Faculty of Medicine, Ain Shams University, Cairo, Egypt; 3 Department of Obstetrics and Gynecology №2, West Kazakhstan Marat Ospanov Medical University, Aktobe, Kazakhstan; 4 Department of Neurology, West Kazakhstan Marat Ospanov Medical University, Aktobe, Kazakhstan; 5 Department of Obstetrics and Gynecology, Faculty of Medicine, Helwan University, Cairo, Egypt

**Keywords:** Calcium, dysmenorrhea, vitamin D, 25-OH Vit. D3: 25-Hydroxyvitamin D3, BMI: Body Mass Index, Ca: Calcium, IU: International Unit, NRS: Numeric Rating Scale, NSAIDs: Non-Steroidal Anti-Inflammatory Drugs, PGDs: Prostaglandins, PTH: Parathyroid Hormone, VAS: Visual Analogue Scale, VDR: Vitamin D Receptor, VIPS: Verbal Intensity Pain Scale, Vit. D: Vitamin D, Vit. E: Vitamin E

## Abstract

Dysmenorrhea, affecting approximately 80% of adolescents, significantly impairs quality of life, disrupts sleep patterns, and induces mood changes. Furthermore, its economic impact is substantial, accounting for an estimated $200 billion in the United States and $4.2 million in Japan annually. This review aimed to identify the effects of vitamin D and calcium on primary dysmenorrhea. We conducted a comprehensive literature search across Web of Science, PubMed, Scopus, and Science Direct, focusing on studies published from 2010 to 2020. Keywords included 'primary dysmenorrhea', 'vitamin D', '25-OH vitamin D3', 'cholecalciferol', and 'calcium'. The quality assessment of the articles was done using the Consolidated Standards of Reporting Trials (CONSORT) and the Strengthening the Reporting of Observational Studies in Epidemiology (STROBE) checklists, and the risk bias was assessed using the Cochrane assessment tool. Abnormal low Vit. D levels increased the severity of primary dysmenorrhea through increased prostaglandins and decreased calcium absorption. Vitamin D and calcium supplements could reduce the severity of primary dysmenorrhea and the need for analgesics. This systematic review found an inverse relation between the severity of dysmenorrhea and low serum Vit. D and calcium.. Vitamin D and calcium supplements could reduce the severity of primary dysmenorrhea and the need for analgesics.

## INTRODUCTION

Dysmenorrhea, characterized by lower abdominal pain occurring just before and sometimes lasting hours after menstruation, often presents alongside symptoms like nausea, vomiting, diarrhea, insomnia, and irritability [1, 2]. This condition affects 80% of adolescents [3-5]. Primary dysmenorrhea occurs in the absence of any pelvic pathology [6], while secondary dysmenorrhea is usually associated with pelvic pathologies (i.e., endometriosis and/or adenomyosis) [7]. Primary dysmenorrhea is primarily attributed to increased local prostaglandin (PGD) levels [8-11], impacting quality of life, sleep patterns, and mood [12]. Dysmenorrhea negatively affects the quality of life and contributes to a disturbed sleep pattern and mood changes [12]. Furthermore, dysmenorrhea imposes a substantial economic burden, costing $200 billion annually in the United States and $4.2 million in Japan [11].

The usual treatment of dysmenorrhea includes non-steroidal anti-inflammatory drugs (NSAIDs) and oral contraceptives [13, 14]. While NSAIDs decrease the severity of dysmenorrhea by inhibiting PGD synthesis, they increase the risk of gastrointestinal bleeding and gastric ulcers [15, 16]. There is little evidence regarding the efficacy of oral contraceptives in the treatment of dysmenorrhea, and 50% of women stopped oral contraceptives prescribed for the treatment of their dysmenorrhea because of their side effects [17].

The treatment of dysmenorrhea with therapeutic options other than NSAIDs and oral contraceptives could be helpful and limit the use of NSAIDs and oral contraceptives. Understanding the presence of vitamin D receptor in the uterus and ovaries [18] highlights the role of Vit. D in regulating inflammatory cytokines [19]. Vit. D metabolites could reduce the level of inflammatory cytokines [20, 21]. Inverse relationships between the severity of dysmenorrhea and serum Vit. D and calcium (Ca) were reported in a systematic review [11]. In addition, Karacin *et al*. [22] found a significant negative association between dysmenorrhea and Vit. D. Kucukceran *et al*. [23] reported a significant reduction in menstrual pain and consumed NSAIDs after a single dose of oral cholecalciferol compared to placebo. A randomized trial reported reduced severity of dysmenorrhea after Vit. D intake [8] and Zarei *et al*. [24] reported a significant reduction in menstrual pain after Ca intake. Consequently, this review aimed to assess the impact of Vit. D and Ca on primary dysmenorrhea.

## MATERIAL AND METHODS

A comprehensive search was conducted across Web of Science, PubMed, Scopus, and Science Direct, focusing on articles/studies published between 2010 and 2020 containing keywords such as "primary dysmenorrhea", "painful menses", and "Vit. D", "Vit. D3", "25-OH Vit. D3", or "cholecalciferol", and "Ca". The objective was to assess the impact of Vit. D and Ca in alleviating the severity of primary dysmenorrhea.

### Inclusion and exclusion criteria

Studies examining vitamin D and/or calcium in primary dysmenorrhea involving non-smoking, non-alcoholic women of reproductive age with regular menses and low serum vitamin D levels and studies that included participants without a history of uterine disorders (i.e., fibroids, adenomyosis, endometrial hyperplasia or endometrial polyps) or ovarian disorders (ovarian cysts, endometriosis, or polycystic ovaries) were included in this systematic review.

Studies that included pregnant women, women with medical disorders (i.e., gastrointestinal, renal, or cardiac disorders), uterine (i.e., fibroids, adenomyosis, endometrial hyperplasia or endometrial polyps), ovarian disorders (ovarian cysts, endometriosis, or polycystic ovaries), women with previous pelvic surgery, psychological or neurological disorders, or who received hormonal therapy were excluded from this systematic review.

### Study selection

**Figure 1:**
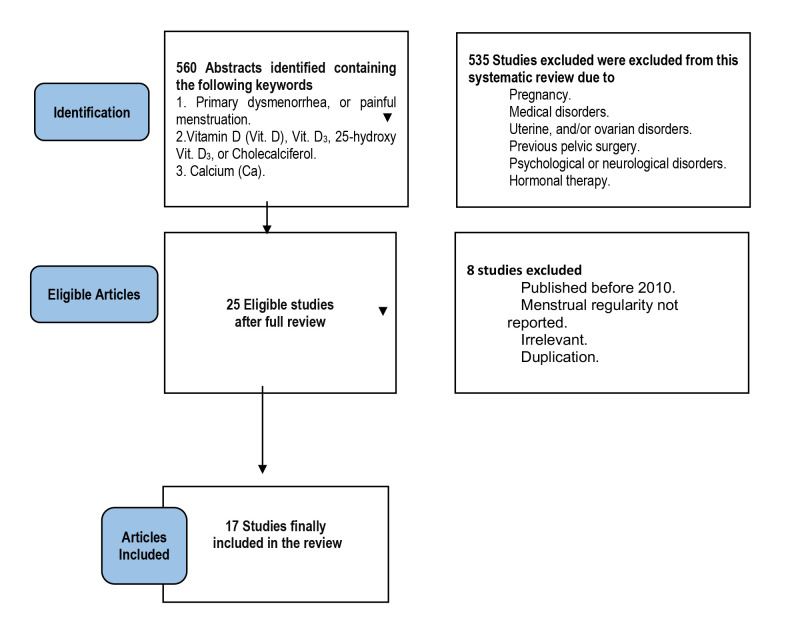
The PRISMA flowchart

Five hundred sixty articles were initially retrieved. Eligible articles were evaluated by two independent authors (AD and IA). After reviewing the titles and abstracts of each article, 535 articles were not eligible for inclusion in this systematic review because of the above-mentioned exclusion criteria (Figure 1). After a full review (i.e., including the results and discussions) of the remaining 25 articles, another 8 articles were excluded (published before 2010, irrelevant or duplicate), and finally, 17 articles were eligible and included in this systematic review (Figure 1).

### Quality assessment

The quality assessment of the articles was done using the Consolidated Standards of Reporting Trials (CONSORT) and the Strengthening the Reporting of Observational Studies in Epidemiology (STROBE) checklists. CONSORT is a 25-item checklist focusing on the article design, analysis, and interpretation. STROBE is a 22-item checklist evaluating different sections of the observational studies [11].

### Risk bias assessment

The risk bias was assessed by two independent authors (AD and IA) using the Cochrane risk-bias assessment tool, which includes selection bias, performance bias, detection bias, attrition bias, and reporting bias.

### Data extraction

The following data were extracted: name of the first author, country, publication year, study design and sample size, age of participants and their age of menarche, body mass index (BMI), duration and severity of dysmenorrhea, intervention, serum Vit. D, dysmenorrhea assessment tool (i.e., visual analog scale [VAS] or numeric rating scale [NRS], or verbal intensity pain scale [VIPS]) and results.

## RESULTS

Out of the 17 studies included in this systematic review, three studies were cross-sectional [25-27], one was a case-control study [22], two were semi-experimental [23, 28], and 11 were randomized controlled studies [24, 29, 30-38]. The studies were conducted in Iran (10 studies) [24-26, 28, 29, 32, 33, 35-37], Turkey (three studies) [22, 23, 30], Jordan (one study) [27], India (1 study) [34], Italy (one study) [38], and Saudi Arabia (one study) [31]. A total of 2,774 participants were included in this systematic review, with most participants having a normal BMI and a normal age of menarche.

### Vitamin D and severity of dysmenorrhea

Although two studies did not find a significant relationship between serum Vit. D levels and the severity of primary dysmenorrhea [25, 27], an inverse association between serum Vit. D and the severity of primary dysmenorrhea was observed in two other studies. These studies reported that individuals with lower serum Vit. D levels experienced more severe primary dysmenorrhea symptoms [22, 26].

### Effectiveness of vitamin D and calcium in reducing the severity of dysmenorrhea

Clinical studies reported that vitamin D supplementation decreased the severity of dysmenorrhea [25, 32-34, 36, 37]. A comparative study [30, 31] found vitamin D more effective than vitamin E or ginger in reducing severe dysmenorrhea symptoms. Participants receiving Vit. D supplements exhibited a higher recovery rate from primary dysmenorrhea and consumed fewer NSAIDs [32]. Zarei *et al*. [24] found that calcium intake was more effective than Vit. D and Ca combined in relieving severe dysmenorrhea. Charandabi *et al*. [33] found that Ca alone or combined with magnesium was equally effective in reducing the severity of dysmenorrhea. Furthermore, Mehrpooya *et al*. [32] reported that omega-3 effectively reduced the pain of primary dysmenorrhea. Table 1 shows the reviewed articles.

**Table 1:** Characteristics of the studies included in the systematic review

Author Country	Design Sample size	Age (yrs.) Menarche age (yrs.)	BMI (Kg/m^2^)	Dysmenorrhea Duration (days)	Intervention (Treatment)	Control	Intensity of dysmenorrhea		Serum Vit. D		Assessment tool	Definition	Results
							Baseline	After	Baseline	After			
Rahnemaie *et al.* [25], Iran	Cross-sectional 143	225±2.97 13.46±1.03	23.53±3.7	2.43±1.32	-	-	6.9±1.59	-	24.45±11.85 ng/mL	-	VAS	≥4	NS
Zeynali *et al.* [26], Iran	Cross-sectional 372	22.4±2.0 13.2±1.44	24±3.07	2.18±1.04	-	-	-Severe 24.73% -Moderate 53.22% -Mild 22.04%	-	-Mild deficiency 37.09% -Moderate deficiency 36.5% -Severe deficiency 26.3%	-	VAS	≥1	S
Karacin *et al.* [22], Turkey	Case- control 368	20.80 12.15	22.26	NM	-	-	7.3±1.4	-	7.1±3.8 ng/mL	-	VAS	1-10	S
Abdul- Razzak *et al.* [27], Jordan	Cross-sectional 56	21.9±2.76 13.6±1.4	NM	NM	-	-	-Very severe 60.7% -Severe 39.3%	-	-Deficient 9% -Insufficient 80% -Normal 11%	-	NRS	0-10	NS
Kucukceran *et al.* [23], Turkey	Quasi experimental 100	20.5 13.1	21.4	2.3	-Insufficient: 7-8 drops Vit. D_3_/day. -Deficient: 9-15 drops Vit. D_3_/day. -Severely deficient: 16–23 drops Vit. D_3_/day.	-	7.0±2.0	4.1±1.6	13.9±6.1	31.1±3.9	VAS	0-10	S
Bahrami *et al.* [28], Iran	Quasi experimental 897	14.7±1.5 12.57±1.19	NM	NM	High-dose cholecalciferol (50,000 IU/week), then 1 capsule over 9 weeks	-	-Mild 9.5% -Moderate 20.8% -Severe 18.1% -Very severe 12.3% -Worst 8.1%	-Mild 11% -Moderate 19% -Severe 16.2% -Very severe 9% -Worst 7.8%	22.7±22.6 nmol/mL	89.9±38.3 nmoL	VIPS	0-5	S
Pakniat *et al.* [29], Iran	RCT 200	22.44±1.9 12.55±1.0	21.62±3.15	2.6±0.89	-Ginger Cap. 500 mg/day + mefenamic 250 mg capsule. -1,000 mg Vit. D + mefenamic 250 mg. -100 IU Vit. E + mefenamic 250 mg for 2 days before and 3 days after menses.	Placebo + Mefenamic 250 mg	7.13±0.8	4.9±1.48	-	-	VAS	0-10	S
Ayşegül *et al.* [30], Turkey	RCT 142	22.0 23.4±5.6	NM	NM	-667 IU of Vit. D/day -200 IU of Vit. E/day for 2 days before and 3 days after the menses.	400 mg Ibuprofen twice a day	8.5±1.2	4.9±2.4	-	-	VAS	0-10	S for Vit. D
Lama *et al.* [31], Saudi-Arabia	RCT 22	13-40 NM	NM	NM	-50,000 IU Vit. D/week + the usual analgesics.	The usual analgesics	7.8	3.6	30.1±13.4 nmol/L	80.2±14.3 nmol	VAS	0-10	S
Zarei *et al.* [24], Iran	RCT 85	23.66 13.1	21.8	NM	-1,000 mg calcium (Ca) + 5,000 IU Vit. D/day -1,000 mg Ca/day from the 15^th^ cycle day until menstrual pain disappears.	Placebo	-Ca/Vit. D: 7.7±1.2 -Ca: 7.7±1.3	-Ca/Vit. D: 5.0±2.6 -Ca: 3.9±2.5	NM	NM	VAS	0-10	S for Ca
Mehrpooya *et al.* [32], Iran	RCT 80	25.24 NM	23.16	NM	Ibuprofen 400 mg as needed. + 1,000 mg Omega-3 every day in the first cycle and 8 days of 2^nd^ and 3^rd^ cycles.	Ibuprofen 400 mg. + 1,000 mg Ca	Omega-3: 6.67±1.8 Ca: 7.5±2.7	Omega-3: 2.3±0.63 Ca: 3.2±1.5	-	-	VAS	≥4	S
Charandabi *et al.* [33], Iran	RCT 61	21.0±2.2 12.6	22.3±3.0	NM	-300 mg Magnesium + 600 mg Ca -600 mg Ca one tab./ day, from the day 15^th^ of their cycle till the day with no menstrual pain.	Placebo	Ca/Mg: 6.0±2.3 Ca:5.2±2.0	Ca/Mg: 3.9±2.1 Ca: 4.2±2.0	-	-	VAS	≥5	S
Fareena *et al.* [34], India	RCT 50	21.18 62% had early onset menarche.	-88% Normal -12% Overweight.	NM	Single oral dose of Vit. D3 3,00,000 for 2-4 months.	Placebo	8.76±0.97	3.56±0.76	17.84±10 ng/mL	34.7±8.1 ng	VAS	0-10	S
Moini *et al.* [35], Iran	RCT 50	26.36 12.72	22.95	NM	50,000 IU oral Vit. D/week	Placebo	7.8	2.8	9.69±5.09 ng/mL	55.4±6.02 ng	VAS	0-10	S
Ataee *et al.* [36], Iran	RCT 54	NM	NM	NM	Single oral dose cholecalciferol (300,000 IU) 5 days before the start of menses for 2 cycles.	Placebo	7.53±1.85	3.77±1.77	7.28±3.64 ng/mL	-	VAS	0-10	S
Zangene *et al.* [37], Iran	RCT 54	22.43 NM	21.03	NM	Single high oral dose Cholecalciferol (300,000 IU) for 5 days before the onset of menses for 3 cycles.	Placebo	7.53±1.85	3.77±1.78	7.37 ng/mL	NM	VAS	0-10	S
Lasco *et al.* [38], Italy	RCT 40	26.65 NM	21.56	NM	Single oral dose cholecalciferol (300,000 IU/mL) for 5 days before the start of menses for 2 cycles.	Placebo	5.85±2.0	3.50±1.27	27.19±7.5 ng/mL	-	VAS	0-10	S
													

BMI: Body mass index. Ca: Calcium. Mg: Magnesium. IU: International unit. NM: Not mentioned. NRS: numeric rating scale. NS: Non-significant. RCT: Randomized controlled trial. S: Significant. VAS: Visual analogue scale. a: verbal intensity pain scale. Vit. D: Vitamin D. Vit. E: Vitamin E.

### Interventions/treatment and duration

The forms of vitamin D used in the clinical studies included in this review were drops or capsules of 100 mg, 667 IU, 50,000 IU, or 300,000 IU. Calcium was used in the form of capsules in clinical studies included in this review. The intervention/treatment duration varied across studies, ranging from 4 to 12 weeks.

### The parathyroid hormone (PTH) and other biochemical markers in dysmenorrhea

Serum Ca and PTH were evaluated in four studies [22, 23, 27, 33]. One study reported significantly lower serum Ca levels and higher PTH levels in the primary dysmenorrhea group compared to controls. In another study, serum Ca and PTH levels were measured in three groups categorized by Vit. D status (insufficient Vit. D (21-29 ng/mL), deficient Vit. D (10-20 ng/mL), and severely deficient Vit. D (<10 ng/mL)), and no significant differences were found in serum Ca and phosphorus levels between the groups before or after Vit. D treatment. In addition, PTH significantly decreased from 42.2±17.4 pg/mL before Vit. D treatment to 28.5±11.0 pg/mL after Vit. D treatment (p<0.001) [23].

Another study investigated vitamin D, calcium, and PTH in adolescents with severe/very severe dysmenorrhea and found that 82.1% of the studied participants had normal serum Ca, 80.4% had normal alkaline phosphatase and 48.2% had hyperparathyroidism [27]. Moini *et al*. [35] measured serum Vit. D, Ca, phosphorus, and alkaline phosphatase in the Vit. D treated group compared to placebo and found that serum Vit. D, phosphorus, and alkaline phosphatase were significantly higher in Vit. D treated group compared to the placebo group.

### Dysmenorrhea-related symptoms

The effect of Vit. D and Ca supplements on the dysmenorrhea-related symptoms were mentioned in five of the reviewed studies [22, 25, 27, 28, 30]. Participants with low serum Vit. D were at greater risk of dysmenorrhea-related symptoms, including headache, fatigue, depression, mood swings, breast tenderness, nausea, and vomiting in two studies [22, 27]. Furthermore, Vit. D intake (high doses) decreased the severity of dysmenorrhea and dysmenorrhea-related symptoms (i.e., backache and crying tendency) [28]. In addition, Mehrpooya *et al*. [32] reported that omega-3 supplementation reduced vomiting and breast tenderness, while calcium intake alleviated bloating symptoms.

Abnormal low serum vitamin D can aggravate the symptoms associated with dysmenorrhea, while Vit. D and Ca intake could improve those symptoms. Low serum vitamin D could increase the severity of primary dysmenorrhea through increased PGD synthesis and decreased intestinal Ca absorption. At the same time, low serum Ca could increase the amplitude of uterine muscle contractility with subsequent uterine muscle ischemia and pain. Consequently, supplementation with Vit. D and Ca may effectively reduce the severity of primary dysmenorrhea and the need for pain-relief medications like NSAIDs.

## DISCUSSION

A comprehensive search was conducted across Web of Science, PubMed, Scopus, and ScienceDirect to retrieve articles and studies published between 2010 and 2020. The search criteria included the following keywords: 1) 'primary dysmenorrhea' or 'painful menses', 2) 'vitamin D', 'vitamin D3', '25-OH vitamin D3', or 'cholecalciferol', 3) 'calcium', with the aim of evaluating the role of vitamin D and calcium in reducing the severity of primary dysmenorrhea. Five hundred sixty articles were initially retrieved. Eligible articles were evaluated by two independent authors (AD and IA). After reviewing the titles and abstracts of each article, 535 of them were not eligible for inclusion in this systematic review (because of the above-mentioned exclusion criteria). After a full review (i.e., including the results and discussions) of the remaining 25 articles, another eight were excluded (published before 2010, irrelevant or duplicate), and finally, 17 were eligible and were included in this systematic review.

A significant relationship between dysmenorrhea and serum Ca was reported by Zarei *et al*. [24]. Impaired Ca regulation is one of the factors contributing to the increased severity of dysmenorrhea [39]. Low serum Ca was also reported in women with premenstrual syndrome (PMS), which supports the role of Ca in neuromuscular regulation [39]. Low serum Ca could increase the amplitude of uterine muscle contraction with subsequent uterine muscle ischemia and pain [40]. The relationship between dysmenorrhea and serum Ca needs further studies.

An inverse relation between the severity of dysmenorrhea and serum Vit. D was reported in Karacin *et al*. [22] and Abdul-Razzak *et al*. [27] studies. Thys-Jacobs [39] also reported Vit. D deficiency in women with dysmenorrhea. Low serum Vit. D increases the severity of primary dysmenorrhea through increased PGD synthesis and decreased intestinal Ca absorption. In addition, it plays a crucial role in Ca absorption and metabolism (stages of hydroxylation) [41].

VDR expression in the uterus and ovaries [18] explains the role of Vit. D in inflammatory cytokine regulation [19]. Vit. D metabolites could reduce the level of inflammatory cytokines [20, 21, 42]. Vitamins, minerals absorption, and metabolism could be important in treating menstrual problems [42].

Abdul-Razzak *et al*. [27] and Anagnostis *et al*. [43] reported an association between severe dysmenorrhea and serum Vit. D and Ca in adolescents.

In addition, a nutritional balance could improve menstrual disorders and dysmenorrhea. Thys-Jacobs [39] reported a close relationship between Ca supplements and reduced severity of dysmenorrhea.

Calcium intake reduces the severity of menstrual cramps and backaches [44]. One study found that menstrual cramps and back pain were reduced after 1,200 mg of Ca per day for three months [40]. Low serum Ca increases uterine cramps and the severity of primary dysmenorrhea [22], which explains the role of Ca in regulating uterine muscle contractions [45].

This systematic review found that Vit. D intake in any dose could effectively reduce the severity of primary dysmenorrhea, and the intake of 50,000 IU of Vit. D weekly is recommended to treat Vit. D deficiency. Vit. D intake may also reduce the risk of PMS, possibly due to the regulation of Ca absorption and inflammatory cytokines [46, 47].

Vit. D changes were also reported with estradiol changes during different phases of the ovulatory and menstrual cycles [39]. A single oral dose (300,000 U) of cholecalciferol for five days before the menstrual flow reduces the severity of primary dysmenorrhea [35]. Vit. D decreases the severity of dysmenorrhea through decreased expression of cyclooxygenase 2 and inhibition of PGD production [48].

This systematic review found a significant positive relationship between the severity of dysmenorrhea and PTH, explained by the role of PTH in renal reabsorption and intestinal absorption of Ca.

The role of Ca in muscle contraction and relaxation was explained previously [47, 48], and the three hormones, calcitonin, PTH, and 25-hydroxy Vit. D (which regulates serum Ca) may play a physiological role in dysmenorrhea [49]. Low Vit. D is often associated with low serum Ca due to decreased intestinal Ca absorption. Low serum Ca increases PTH secretion with a subsequent increase in the renal reabsorption and intestinal absorption of Ca [48].

No significant relationship between serum phosphorus and primary dysmenorrhea was found in this systematic review, which needs to be confirmed in future studies. However, we found a significant relationship between the severity of dysmenorrhea-related symptoms and both serum Vit. D and Ca. Additionally, this review found that Vit. D and Ca supplements could reduce primary dysmenorrhea and the consumed analgesics.

Bertone-Johnson *et al*. [46] and Baird *et al*. [50] reported an inverse relationship between serum Vit. D and the risk of dysmenorrhea and mood changes. Rahnemaie *et al*. [25], found that serum levels of Vit. D was inversely related to the severity of dysmenorrhea-associated symptoms, including fatigue, headache, nausea, and vomiting.

Research on the impact of fish oil on primary dysmenorrhea is limited [51]. However, a study by Zamani *et al*. [52] indicated that fish oil intake can reduce the severity of primary dysmenorrhea. This effect is likely due to the ability of fish oil to inhibit the production of PGDs and leukotrienes, which are known to contribute to menstrual pain. Vitamin E and omega-3 intake have been observed to lessen the severity of dysmenorrhea. This reduction in pain severity may be attributed to the stimulating effect of vitamin E on beta-endorphins, which are natural pain-relieving compounds in the body [53].

Daily *et al*. [54] found that vitamin D, E, and ginger effectively decreased the severity of dysmenorrhea (the effect was more favorable in the ginger group than in the vitamin D and E groups). Rahnama *et al*. [11] suggested that ginger contents (i.e., gingerol and gingerdione) may have analgesic and anti-inflammatory effects [55]. Further, in-vitro studies support this by showing that ginger can inhibit the production of PGDs and leukotrienes, which are known to exacerbate menstrual pain [29]. The limited number of research investigating the effects of vitamin D and calcium on the severity of dysmenorrhea was the only limitation of this study, and further studies in this area are warranted.

## CONCLUSION

This systematic review found an inverse relationship between the severity of dysmenorrhea and low serum levels of vitamin D and calcium. The findings suggest that supplementation with vitamin D and calcium could effectively reduce the severity of primary dysmenorrhea and the reliance on analgesics.
